# The Role of Physiotherapy in Peripheral Artery Disease in Patients With Diabetes Mellitus: A Narrative Review

**DOI:** 10.7759/cureus.52019

**Published:** 2024-01-10

**Authors:** Domenica Herrera, Diego E Rueda Capistrani, Sebastian Obando Vera, Camila Sanchez Cruz, Karal A Linarez Nuñez, Douglas Banegas, Ariane Argueta, Maria Isabel Murillo, MD, Kenol Clervil, Elda J Perez Moreno, Ernesto Calderon Martinez

**Affiliations:** 1 Medicine, Pontificia Universidad Católica del Ecuador, Quito, ECU; 2 Physiotherapy, Universidad Nacional Autonoma de Mexico, Ciudad de Mexico, MEX; 3 General Medicine, Universidad Catolica de Santa Maria, Arequipa, PER; 4 General Practice, Universidad Nacional Autonoma de Mexico, Ciudad de Mexico, MEX; 5 General Medicine, Universidad Catolica de Honduras, Tegucigalpa, HND; 6 General Medicine, Universidad Nacional Autonoma de Honduras, San Pedro Sula, HND; 7 Medicine, Universidad Salvadoreña Alberto Maferrer, San Salvador, SLV; 8 Primary Care, Universidad Católica de Honduras, Tegucigalpa, HND; 9 Emergency Department, Instituto Tecnologico de Santo Domingo (INTEC), Charlotte, USA; 10 Digital Health, Universidad Nacional Autónoma de México, Ciudad de Mexico, MEX

**Keywords:** dm, ankle brachial index., physiotherapy, peripheral artery disease, diabetes

## Abstract

Diabetes mellitus (DM) comprises a spectrum of metabolic disorders distinguished by the persistent elevation of glucose levels in the bloodstream. It stands as a primary risk factor for peripheral arterial disease (PAD), denoted by atherosclerosis affecting the lower extremities. One clinical manifestation of symptomatic PAD is intermittent claudication alleviated by rest but also capable of presenting as atypical leg pain. Confirmatory diagnostic measures, including the ankle-brachial index (ABI), toe-brachial index (TBI), or Doppler waveform analysis, are imperative in the verification of PAD. For management, the recommendation is to incorporate physiotherapy alongside concurrent medical interventions, such as anticoagulants, antiplatelet agents, statins, or, in certain cases, surgical procedures. This narrative review seeks to elucidate the advantages of physiotherapy in diabetic patients with PAD, contributing to the deceleration of disease progression and improving symptoms. Although supervised exercise therapy is strongly supported by empirical evidence as more beneficial, the absence of supervised environments is a common issue. Consequently, the preference lies in the combination of supervised exercise with home-based regimens. The objective is that each patient exercises for more than three days per week, progressively extending their duration weekly. This approach has demonstrated a noteworthy enhancement in walking functionality, exercise tolerance, pain alleviation, and an overall improvement in the quality of life for patients.

## Introduction and background

Diabetes mellitus (DM) is a group of metabolic diseases characterized by chronic hyperglycemia [1,2]. It stands as a significant risk factor for various forms of cardiovascular diseases and is the leading cause of mortality among adults with DM [3,4]. DM represents a strong risk factor for peripheral artery disease (PAD), described as atherosclerosis of the lower extremities [5,6]. PAD's global prevalence extends to over 200 million individuals, with approximately 8.5 million affected in the United States [7,8]. Although the prevalence of PAD varies widely, approximately 20% of adults older than 55 have PAD [8,9]. One-third of patients with PAD have DM, the prevalence of PAD in these patients may be underestimated due to the asymptomatic nature of less severe PAD and concomitant diabetic neuropathy [10,11].

The underlying metabolic abnormalities in DM enhance vascular inflammation, endothelial dysfunction, platelet activation, and hypercoagulation, processes important to the pathogenesis of PAD [12]. Strong evidence shows an increased risk of death in patients with DM and PAD, at least two times compared with the normal population [13,14]. Additionally, firm evidence shows that diabetes-related patients with PAD are five times more likely than non-diabetes-related patients with PAD to have an amputation [15]. Even though approximately half of individuals with PAD do not display any symptoms or have unusual ones, the most common indication for mild to moderate PAD is intermittent claudication; this is characterized by pain, cramping, aching, tiredness, or heaviness that occurs in one or both legs (usually in the calves) while walking and does not subside with continued walking but is alleviated by rest [16]. At least 5% of Americans over 55 are affected by claudication, and this condition is present in 15-40% of patients who have PAD [17]. The major goal of contemporary treatment of claudication is to improve walking ability and functional status by reducing claudication symptoms and preserving limb viability. The treatment usually consists of exercise training, pharmacotherapy, and revascularization. Among these, exercise training programs are now recommended as an initial intervention, although the precise role of these programs in patient recovery is still not entirely elucidated. This study thus seeks to provide a comprehensive review of the existing literature, with the goal of better understanding the specific contributions of physiotherapy in the context of patients living with both PAD and DM.

## Review

Peripheral artery disease

PAD is defined as atherosclerosis of the arteries in the lower extremities [6,18]. It is associated with reduced exercise capacity and a decreased quality of life [19,20]. Several classification systems for PAD exist, including the Fontaine Classification [21], the Rutherford Classification [22], the Global Limb Anatomic Staging System (GLASS) [23], and the Trans-Atlantic Inter-Society Consensus (TASC) [24]. While other disease processes can lead to the narrowing of limb arteries and symptoms of arterial insufficiency, atherosclerosis remains the most prevalent etiology [25,26]. Well-defined risk factors are associated with the development of PAD, including smoking, DM, hypertension, and hypercholesterolemia, with smoking and DM being the most predominant ones [18,27]. PAD is cited historically as more prevalent in males than females [28,29]. However, recent studies indicate that the prevalence of PAD in females is at least as high as in males across all age groups and even greater in females over the age of 70 compared to males of the same age [30,31].

Most symptomatic PAD patients present with lower extremity pain, either as classic intermittent claudication or atypical leg pain [17,32]. The clinical manifestations of PAD predominantly result from progressive luminal narrowing, leading to claudication, rest pain, ulceration, and gangrene. Chronic limb-threatening ischemia (CLTI) is a clinical syndrome defined by PAD in combination with rest pain or tissue loss (ulceration or gangrene) lasting more than two weeks. CLTI is the preferred term to replace critical limb ischemia or severe limb ischemia. Both rest pain and tissue loss represent different states in CLTI and have diverging prognoses [23].

Confirmation of PAD requires diagnostic testing. The resting ankle-brachial index (ABI) is a simple, noninvasive test that measures systolic blood pressures at the arms and ankles in the supine position using a Doppler device; this procedure can be performed with new point-of-care ultrasonography (POCUS) devices but require learning [30,33-35]. An ABI ≤0.90 is sensitive and specific for arterial stenosis and is diagnostic for PAD [36,37]. Arterial wall calcification can potentially lead to a falsely elevated ABI in individuals with DM and other comorbidities [37,38]. In such cases, other noninvasive tests, such as toe-brachial index (TBI) measurement or Doppler waveform analysis, may help detect occlusive disease [37,38]. Patients with PAD who have a definitive history of claudication and those with atypical extremity pain might have a normal resting ABI. For these patients, exercise testing is indicated [39,40]. An exercise therapy program is recommended as part of the initial treatment for symptomatic patients with PAD, as evidence demonstrates significant improvements in walking parameters [41,42]. Regardless of the mode of delivery, all exercise programs should be progressive and individually prescribed when possible [43]. Supervised exercise training (SET) appears to be the better initial approach [44,45]. The accessibility of supervised exercise training programs for PAD is limited [46]. Home-based exercise may be a helpful alternative for PAD patients when SET is not available as the primary treatment option [46,47].

Diabetes mellitus

DM is a metabolic disease that results in hyperglycemia from abnormal carbohydrate metabolism due to insulin deficiency, peripheral insulin resistance, or both [1,48]. About 422 million people worldwide have DM, and over a million deaths are directly attributed to this disease each year [49]. As of January 2022, according to The National Diabetes Report, over 30 million people have diabetes, and over 90 million have prediabetes in the United States [50]. T2DM and the vascular complications of this disease continue to increase in prevalence incidence and are a leading cause of human suffering and death [51]. The etiology of DM is multifactorial and involves a complex combination of genetics and environmental factors. Strong evidence shows that lifestyle modifications, such as healthy eating and exercise, can reduce or delay the incidence of diabetes [52,53].

PAD and diabetes

**Figure 1:**
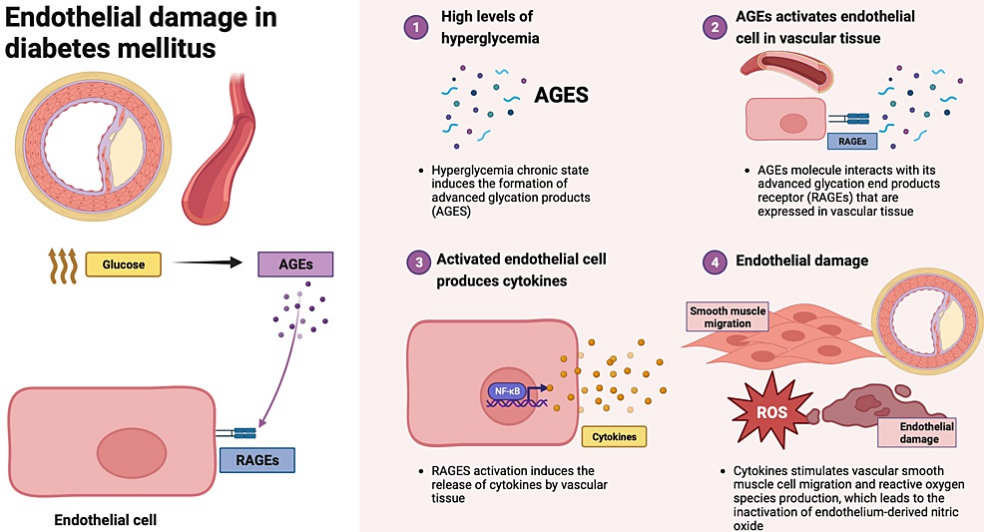
Endothelial damage in diabetes mellitus 1. Hyperglycemia induces the formation of advanced glycation products (AGEs). 2. AGE molecules interact with their advanced glycation end-product receptors (RAGEs) expressed in vascular tissue. 3. RAGE activation induces the release of cytokines by endothelial cells. 4. Cytokines stimulate vascular smooth muscle cell migration and reactive oxygen species production. 5. Finally, this cascade of events leads to the inactivation of endothelium-derived nitric oxide, and therefore, endothelial damage and loss of function of the vascular tissue. Credits to: Domenica Herrera 2023. Created with BioRender

An atherogenic process triggers PAD in a debilitated vessel wall that induces progressive narrowing, blockade, and, therefore, ischemia of peripheral arteries [54,55]. A significant body of evidence suggests that patients with diabetes patients have an increased predisposition to PAD, which tends to occur early with accelerated progression and is frequently asymptomatic [55,56]. Patients with diabetes patients have twice the risk of developing PAD than those without DM [57]. This association is better explained due to an overproduction of advanced glycation end products, a chronic state of inflammation, and oxidative stress [55,56]. This process is illustrated in Figure 1 [55-57].

Recent evidence suggests that higher plasma levels of RAGEs are associated with increased risk of amputation (i.e., decreased amputation-free survival) in type 2 DM-related subjects [56,57]. Furthermore, this evidence suggests that S100-proteins, a family of endogenous proinflammatory proteins, play a role in overall PAD mortality by activating the RAGE system [57]. In addition, the unregulated uptake of low-density protein (ox-LDL) by the vascular wall is crucial in the progression of atherosclerosis [58]. One of the most novel markers for peripheral artery disease is the lectin-like oxidized LDL receptor 1 (LOX-1). Recent evidence demonstrates that high serum levels of LOX-1 in patients with DM are independently associated with PAD [56,58]. Medial arterial calcification (MAC) is characterized by arterial stiffness and is frequently associated with DM. MAC is mediated by the accumulation of calcium phosphate with hydroxyapatite formation due to the overproduction of ROS in DM. Evidence suggests a strong association between below-the-knee MAC and lower limb amputation risk [55,59].

Physiotherapy intervention

Intermittent claudication is the most common symptom of PAD and leads to gait deviation accompanied by pain that promotes a sedentary lifestyle, accelerated deterioration, reduced strength, and muscular endurance [47,60]. For this reason, rehabilitation is crucial for effective PAD treatment; therapies aim to slow the disease progression and stimulate collateral circulation to counteract insufficient perfusion [61].

Molecular mechanism

Several laboratory studies have demonstrated that blood flow is not the sole determinant of muscle function in PAD patients [62,63]. Inflammation and oxidative stress lead to accelerated myopathy by damaging mitochondrial electron transport chain function, thereby reducing energy production and increasing apoptosis and sarcopenia [64]. During physical exercise, muscle contractions stimulate active metabolism through an insulin-independent pathway to increase glucose uptake by muscle [61,65]. Glycosylated hemoglobin is an index of long-term glycemic control and has shown improvement by regular exercise. Therefore, aerobic and muscular resistance exercises are recommended to achieve better glycemic control and are the first-line treatment in patients with intermittent claudication due to PAD [42,66]. Moreover, evidence has shown that physical training increases the expression of exercise-induced antioxidant enzymes such as glutathione peroxide dismutase, glycation reductase, and catalase [67].

Higher levels of community-based daily ambulatory activity are associated with higher levels of circulating antioxidant capacity, lower levels of inflammation determined by high-sensitivity C-reactive protein, and no increase in oxidative stress markers in symptomatic patients with PAD [64].

Types of therapy

Numerous studies have demonstrated different therapies and interventions to have an impact on our specific population to increase the pain-free walking distance in patients with PAD [24,68,69].

**Table 1:** Most relevant articles identified for the role of physiotherapy in PAD in patients with DM IC: Intermittent Claudication; PTA: Percutaneous Transluminal Angioplasty; SET: Supervised Exercise Therapy

Authors	Year	Country	Number of participants	Results
Castro-Sánchez et al. [68]	2013	Spain	68	Improvement in ABI, Doppler flow velocity, and blood parameters in patients with type 2 diabetes with physical therapy.
Kim M. van Pul et al. [70]	2012	The Netherlands	775	Supervised exercise therapy (SET) for patients with intermittent claudication is equally effective in improving walking distance for patients with and without DM, although absolute claudication distance remains lower in patients with DM.
Schlager, Oliver et al. [71]	2011	Austria	40	Six months after training cessation, the beneficial effect of SET on EPC (Endothelial Progenitor Cells) diminished, but maximum walking distance was significantly improved compared to baseline and controls.
Murphy, Timothy P. et al. [72]	2012	United States of America and Canada	119	This study demonstrates that for patients with claudication, SE provides a superior improvement in treadmill walking performance compared to both primary aortoiliac ST and Optimal Medical Care (OMC: home walking and cilostazol) over 6 months.
Mary O Whipple et al. [73]	2020	United States	44	Participants with diabetes experienced greater improvements in claudication onset distance with exercise.
Oakley C et al. [74]	2008	United Kindom	20	Nordic pole enables patients with intermittent claudication to walk further with less pain, despite a higher workload.
Edwing Langbein et al. [75]	2002	United States	52	Polestriding improved exercise tolerance on the constant work rate and incremental treadmill tests.
Makoto Haga et al. [76]	2020	Japan	16	Bicycle exercise training improved the quality of life and walking distance.

Supervised interval treadmill training is established as a first-line therapy in claudication treatment programs, including for those with diabetes [77,78]. The principles of supervised exercise training (SET) are guided by the 2007 TASC II guidelines [28] and updated by the European Society of Cardiology (ESC) in collaboration with the European Society for Vascular Surgery (ESVS) [79]. Exercise sessions should last 30-60 minutes with breaks before reaching maximal claudication distance and be conducted three to five times per week for three to six months. Educating patients about the non-harmful nature of ischemic pain during exercise and the importance of foot care post-session is vital [47,77,78,80,81]. A systematic review focused on the impact of diabetes as a comorbidity in patients with intermittent claudication and how it affects the outcomes of SET showed improved walking performance in both DM and non-DM patients [82].

Firm evidence supports the necessity of supervised exercise for optimal results, as general physician recommendations may not result in clinical benefit [24,83]. Nevertheless, the lack of supervised settings for exercise rehabilitation is a major barrier to patient referrals and the lack of patient motivation [84]. To maximize the effectiveness of rehabilitation programs, many healthcare providers combine supervised outpatient sessions with unsupervised programs (home exercise programs). This approach attempts to ensure that patients receive professional guidance, support, and the flexibility to continue their exercises independently. Such programs often prioritize regularity over intensity, as consistent exercise is believed to have a more significant overall impact on patient recovery [26], despite this, some recent meta-analyses showed that high-intensity interval training (HIIT) can have benefits in claudication in the short term while low-intensity training will require more time to have the same results [85].

Home-based exercise

Additionally, effective programs of structured home-based exercise have advised patients with PAD to walk for exercise three to five times per week and to walk to maximize ischemic leg pain. Patients with PAD who are advised to engage in home-based exercise should be instructed to write down walking exercise goals and to record their walking exercise activity each week to better monitor patients and dose physical exercise. These programs should be individualized. Starting as little as 10 minutes for walking per session and increasing walking per session by 5 minutes per week until patients are walking for exercise 45 to 50 minutes per session to progressively increase walking resistance before the onset of intermittent claudication or pain [86-88]. The degree of benefit of supervised and home-based exercise programs has not yet been demonstrated [89,90].

Supervised exercise program

Firm evidence suggests as first-line therapy a supervised exercise program consisting of walking a minimum of 3 times a week (30-60 minutes/session) for at least 12 weeks (about three months) [39,89,91,92]. It is performed on a treadmill with increasing exercise intensity, at a speed and degree that induces moderate claudication within 3-5 minutes, after which the patient stops the exercise, recovers, and the cycle is restarted once the symptoms are resolved [93-95]. Currently, treadmill interval walking is suggested as the main treatment [39,96]. However, not all patients with PAD can perform them; for example, those who present intermittent claudication because they report pain [39,96]. The patients are recommended to follow the same criteria as home-based exercise, starting as little as 10 minutes for walking per session and increasing walking per session by 5 minutes per week until patients are walking for exercise 45 to 50 minutes per session [88,97,98].

Recommendations

In patients with claudication, a supervised exercise program is recommended to enhance functional status and improve the quality of life while reducing leg symptoms [92]. For cases where supervised exercise programs are unavailable, home-based exercise is encouraged, with a target of at least 30 minutes of walking three to five times per week, which proves particularly beneficial for claudication patients [39]. Furthermore, for individuals experiencing claudication accompanied by pain, alternative activities, such as upper body ergometry, cycling, and pain-free or low-intensity walking, are suggested to enhance gait ability and functionality [99,100]. In the case of patients with PAD, a structured exercise program, whether in a community or home setting and incorporating behavior change techniques, may offer significant advantages in improving walking ability and functionality, as well as promoting social inclusion [47,101]. Additionally, for patients with impaired gait, a supervised exercise program is recommended to enhance functionality, improve the quality of life, and alleviate leg symptoms [93]. Strong evidence indicates that resistance exercise can lead to improved gait, and exercise programs have demonstrated the potential to increase the distance from the onset to maximum claudication pain [102,103].

**Figure 2:**
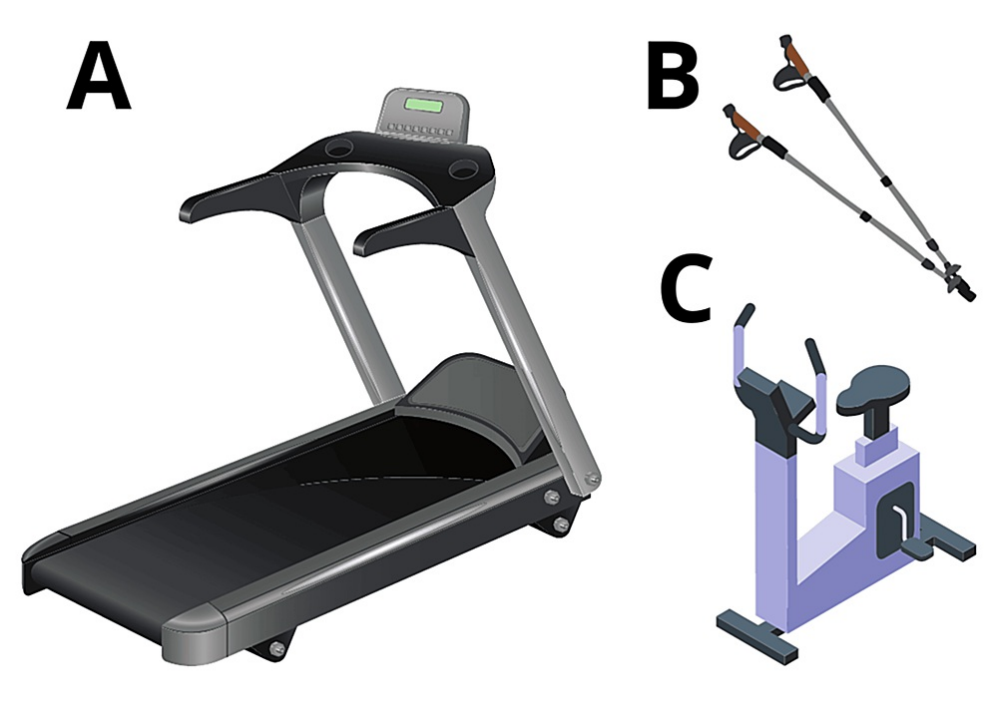
Most common devices used on physiotherapy patients with diabetes and PAD A) Treadmill; B) Nordic pole; C) Cycloergometer PAD: Percutaneous Transluminal Angioplasty

Nordic pole walking (NPW) and cycloergometer (Figure 2) exercise have been recognized as valuable complementary interventions for patients with peripheral artery disease (PAD), particularly those also managing diabetes mellitus (DM). NPW, in particular, has gained prominence as a recommended exercise for cardiovascular patients due to its unique method of engaging both the arms and trunk muscles. This full-body engagement is beneficial because it significantly reduces the load on the lower limbs [74,104]. By distributing the physical effort across more muscle groups, NPW not only helps in improving overall exercise tolerance but also positively impacts the quality of life for patients. Furthermore, NPW has been shown to decrease claudication pain - a common symptom in PAD patients characterized by cramping pain in the legs due to inadequate blood flow during exercise. Importantly, studies have demonstrated that NPW can significantly increase the maximal walking distance when compared to traditional treadmill training [74,75,104]. This increase indicates that patients are able to walk longer distances before experiencing claudication pain, thus improving their functional capacity and endurance. Cycloergometer exercise, on the other hand, offers a beneficial alternative for PAD patients who find treadmill exercises to be too strenuous or uncomfortable, particularly for those with DM. The design and nature of cycloergometer exercise, which involves stationary cycling, make it a less demanding form of exercise on the body’s joints and spine [76]. This feature is especially advantageous for elderly and obese patients, as it minimizes the risk of joint-related injuries or discomfort that can occur with weight-bearing exercises like walking or running.

Contraindications

Evidence shows that exercise is contraindicated in patients with acute coronary disease until the condition stabilizes after approximately five days. Other contraindications include aortic stenosis, dyspnea at rest, myocarditis, endocarditis, pericarditis, fever, and uncontrolled arterial hypertension [105,106]. Regular exercise is recommended for patients with coronary artery disease after graded exercise testing to rule out and treat unstable angina or ischemia [106,107].

Other therapies 

Pneumatic Compression

Intermittent and pulsatile pneumatic compression therapy is an important adjunct treatment for patients with PAD, especially those also managing diabetes [108]. This therapy involves the application of external pressure to the extremities using a pump that is periodically inflated. It operates by transferring pressure in a controlled manner, enhancing blood flow, and promoting circulation. This process is beneficial in several ways. First, it enhances nitric oxide release, which is crucial for vasodilation, improving the flow of blood through the arteries [108]. Second, it improves overall perfusion, ensuring that blood reaches even those areas of the limbs where circulation is typically compromised due to PAD. Lastly, it helps in reducing tissue edema, a common issue in PAD patients, by encouraging fluid movement out of the swollen tissues [108].

One of the significant advantages of this therapy is its effectiveness in healing wounds and reducing chronic pain, particularly in patients suffering from critical limb ischemia (CLI). Additionally, it has been found to improve walking distances in patients with intermittent claudication [109,110]. Another key aspect of this therapy is its versatility and safety, making it suitable for both hospital and outpatient settings. With appropriate training, patients can even administer this therapy at home, providing a convenient and accessible treatment option [109,110]

Hyperbaric Oxygen Therapy

Hyperbaric oxygen therapy (HOT) presents another therapeutic approach for patients with PAD and diabetes. This therapy involves exposing the patient to 100% oxygen at high pressure in a specialized chamber. HOT has several physiological effects that are beneficial for diabetic patients, such as increasing the ability of neutrophils to kill bacteria, stimulating angiogenesis (the formation of new blood vessels), and enhancing fibroblast activity and collagen synthesis due to the high-pressure environment that allows oxygen to dissolve into the plasma more effectively, increasing its reach to tissues with poor perfusion [111,112]. These effects are particularly important for diabetic populations prone to infections and complications from PAD. The efficacy of HOT, however, is a subject of ongoing debate. While some studies indicate no significant improvement in healing ischemic ulcers, others have reported its effectiveness in treating infected diabetic feet [111,112]. A 2021 meta-analysis suggested potential benefits in improving the healing of diabetic foot ulcers and reducing rates of major lower extremity amputations, though it calls for more comprehensive trials to solidify these findings [113].

Functional Electrical Stimulation

Functional electrical stimulation (FES) is increasingly recognized as a beneficial adjunct therapy for individuals with intermittent claudication [114,115]. In this condition, often linked to PAD, the primary therapeutic approach for managing intermittent claudication is physical exercise, which enhances circulation and muscle strength. FES involves the application of electrical currents to the muscles, mimicking the contractions that occur during physical activity. This method can improve blood flow, alleviate pain, and augment muscle function, making it particularly valuable for patients who may struggle with regular exercise. A recent systematic review has found that there is not enough evidence to suggest a clinical decision based on the efficacy of electrical stimulation for the management of impaired walking function in patients with PAD [116]. The combination of physical exercise and FES has been shown to be more effective, as indicated by the physiological benefits observed in the circulatory system in studies [64,101].

Lower Limb Offloading

Lower limb offloading is a crucial strategy, particularly for patients with a neuropathic foot, assisting in the treatment of ischemic ulcers [117-119]. Offloading aims to reduce pressure and stress on the affected areas, allowing ulcers and wounds to heal. This can be achieved through various methods, depending on the patient's characteristics, ability, and acceptance. Shoe modifications and tailored insoles can redistribute pressure away from ulcers. Podological treatments provide specialized foot care while special plaster dressings protect and support healing ulcers. In more severe cases, using crutches or a wheelchair might be necessary to completely offload the lower limb [117-119]. These offloading strategies are an integral part of the comprehensive care plan for patients with PAD and diabetes, aimed at minimizing complications and promoting healing.​​​​​​

## Conclusions

In conclusion, peripheral artery disease (PAD) and diabetes mellitus (DM) present significant challenges in the medical field due to their complex interplay and the severe impact they have on patients' quality of life. Management of these conditions involves a multifaceted approach. The mainstay of treatment for PAD, particularly when presenting as intermittent claudication, is exercise therapy. Supervised exercise training (SET) is recognized as an effective first-line therapy, with guidelines suggesting sessions of 30-60 minutes, 3-5 times a week. For patients unable to access SET, home-based exercise programs serve as a viable alternative, emphasizing the importance of regular, progressive walking exercises. Furthermore, adjunct therapies, such as pneumatic compression and hyperbaric oxygen therapy (HOT) offer additional benefits in managing PAD, especially in diabetic patients. These therapies enhance blood flow, promote wound healing, and can alleviate chronic pain. In cases where PAD is complicated by diabetic foot ulcers, strategies like lower limb offloading are crucial for effective treatment.

Lastly, the role of functional electrical stimulation (FES) in managing intermittent claudication in PAD patients is emerging as a promising adjunctive treatment. Though its clinical efficacy requires further validation, it shows the potential to enhance the benefits of physical exercise. Overall, the management of PAD and DM necessitates a holistic approach, combining physical therapy, lifestyle modifications, and innovative therapeutic strategies to improve patient outcomes.
